# Supplementary material for: The Asanté™ HIV-1 Rapid Recency® Assay is reliable, feasible, and acceptable for use at the point-of-care in Lusaka, Zambia
Source: PLoS One. 2026 May 4;21(5):e0346868. doi: 10.1371/journal.pone.0346868 (PMC13138624; doi:10.1371/journal.pone.0346868)
Supplement: S1 File — (DOCX) [file pone.0346868.s001.docx]

**Supporting information file 1: supplementary tables and figures**

**Title**: The *Asanté*™ HIV-1 *Rapid Recency*® Assay is reliable, feasible, and acceptable for use at the point-of-care in Lusaka, Zambia.

**Authors:** Shilpa S. Iyer^1^, Jake M. Pry^1,2*^, Herbert Kapesa^1^, Misinzo Moono^1^, Chilambwe Mwila^1^, Christiana Frimpong^1^, Mirriam Nanyangwe^1^, Lumbani Phiri^1^, Ruth Ngandu^1^, Precious Sakanya^1^, Sharon Mwansa^1^, Talandila Phiri^1^, Mizinga Haciwa^1^, Patricia Maritim^1^, Kemba Lee^3^, Melissa Arons^3^, Tiffiany Aholou^3^, Peter Minchella^4^, Theodora Savory^1^, Carolyn Bolton^1,5^ and Michael E. Herce^1,6^

**Affiliations**:

^1^Centre for Infectious Disease Research in Zambia, Lusaka, Zambia

^2^University of California, Davis, California, USA

^3^Division of Global HIV & TB, Global Health Center, Centers for Disease Control and Prevention, Atlanta, Georgia, USA

^4^Division of Global HIV & TB, Global Health Center, Centers for Disease Control and Prevention, Namibia, Windhoek, Namibia

^5^University of Alabama at Birmingham, Alabama, USA

^6^University of North Carolina, Chapel Hill, North Carolina, USA

**Index:**

- *Figure S1*: Receiver-operator curve for Asanté test conducted at point-of-care and central laboratory compared to RITA results, excluding participants with suppressed baseline viral load (N=182).
- *Table S1*: Rapid test for recent infection classification characteristics by test setting compared to the Zambia national program recent infection testing algorithm as the reference standard, excluding participants with suppressed baseline viral load (N=182).
- *Figure S2*: Scatter plot of baseline viral load result by enrolment date and RITA status.
- *Table S2*: Two-by-two table showing point-of-care Asanté results compared to RITA results
- *Table S3*: Two-by-two table showing central laboratory Asanté results compared to RITA results

**Figure S1**: Receiver-operator curve for Asanté test conducted at point-of-care and central laboratory compared to RITA results, excluding participants with suppressed baseline viral load (N=182).


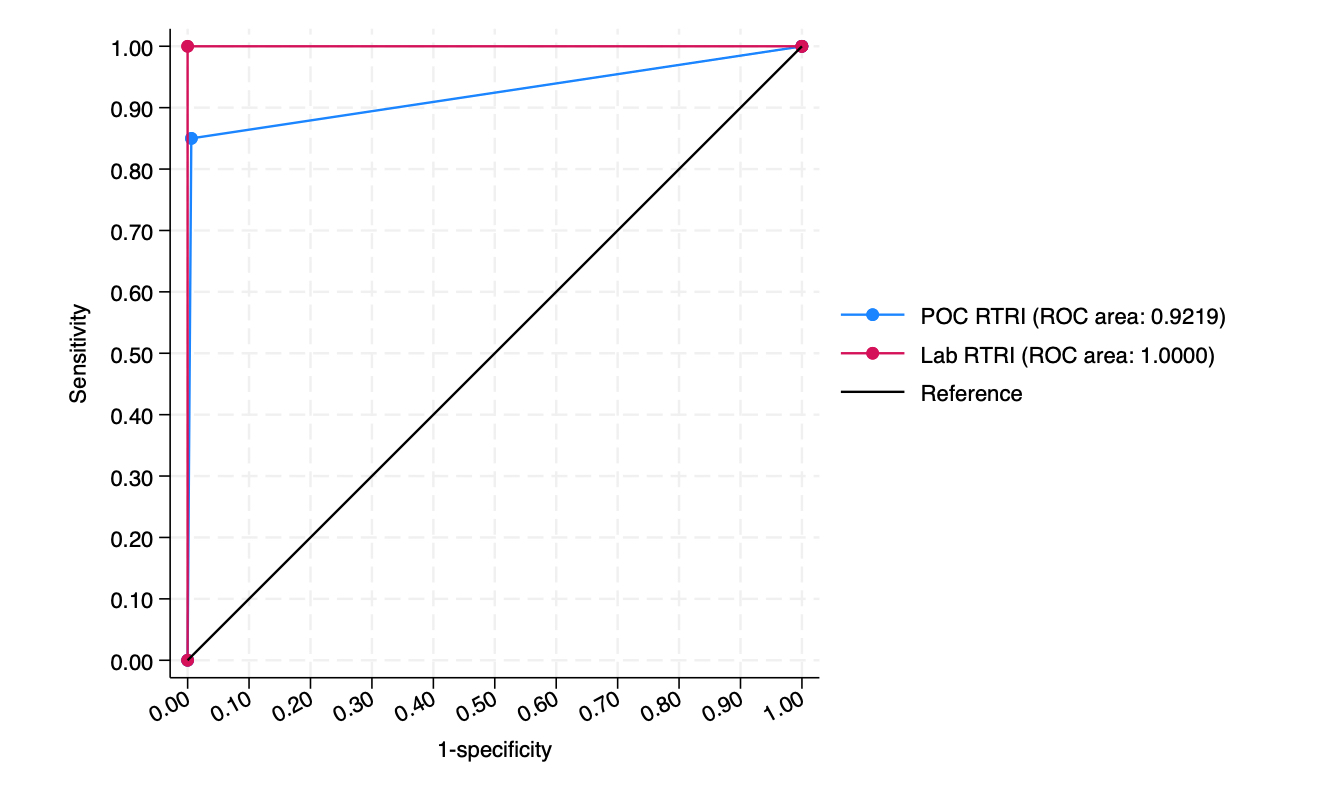


Note: RTRI – rapid test for recent infection, POC – point of care, ROC – receiver-operator curve, CL – central laboratory

**Table S1**: Rapid test for recent infection classification characteristics by test setting compared to the Zambia national program recent infection testing algorithm as the reference standard, excluding participants with suppressed baseline viral load (N=182).

| *RTRI test setting* | *Sensitivity compared to RITA* | *Specificity*  *Compared to RITA* | *Correctly classified* |
| --- | --- | --- | --- |
| POC | 85.0% | 99.4% | 97.8% |
| CL | 100.0% | 100.0% | 100.0% |

Note: RTRI – rapid test for recent infection, CL – central laboratory, POC – point-of-care, RITA- recent infection testing algorithm

**Figure S2**: Scatter plot of baseline viral load result by enrolment date and RITA status.


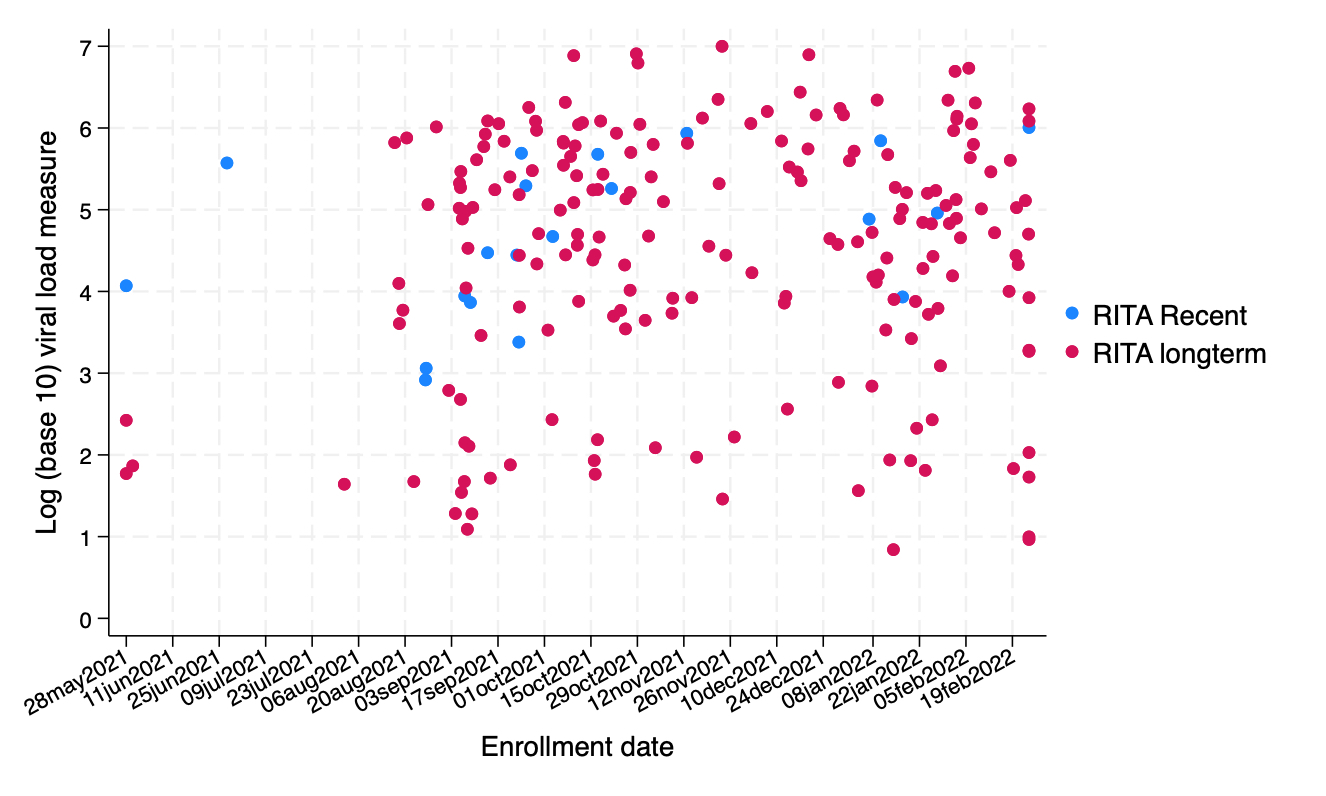


**Table S2**: Two-by-two table showing point-of-care Asanté results compared to RITA results

|  |  | RITA result | |
| --- | --- | --- | --- |
|  |  | + | - |
| POC result | + | 213 | 3 |
|  | - | 12 | 17 |
| RITA - recent infection testing algorithm | | | |

Note: POC – point of care, RITA – recent infection testing algorithm

**Table S3**: Two-by-two table showing central laboratory Asanté results compared to RITA results

|  |  | RITA result | |
| --- | --- | --- | --- |
|  |  | + | - |
| CL result | + | 210 | 0 |
|  | - | 15 | 20 |
| RITA - recent infection testing algorithm | | | |

Note: CL – central laboratory, RITA – recent infection testing algorithm
