# Supplementary material for: The Asanté™ HIV-1 Rapid Recency® Assay is reliable, feasible, and acceptable for use at the point-of-care in Lusaka, Zambia
Source: PLoS One. 2026 May 4;21(5):e0346868. doi: 10.1371/journal.pone.0346868 (PMC13138624; doi:10.1371/journal.pone.0346868)
Supplement: Attachment — Submitted filename: PONE_D-25_63413_Reviewer Comments.docx [file pone.0346868.s002.docx]

**Manuscript ID:** PONE_D-25-63413

**Manuscript Title:** The Asanté™ HIV-1 Rapid Recency® Assay is reliable, feasible, and acceptable for

use at the point-of-care in Lusaka, Zambia.

**Recommendation: Accept with minor revisions.** The manuscript provides valuable evidence on the field performance of the Asant HIV-1 RTRI at POC in Zambia.

This is a valuable and timely study that addresses an important gap in HIV recency testing at point-of-care in a resource-limited setting. The manuscript presents a robust mixed-methods evaluation of the Asant HIV-1 Rapid Recency Assay (RTRI) at point-of-care (POC) in Lusaka, Zambia, demonstrating strong concordance with Central Laboratory testing and feasibility among healthcare workers. Minor revisions are needed to enhance clarity, generalizability, and methodological precision, thereby improving readiness for publication.

Comments:

General: The author(s) declare in the financial disclosure section that no specific funding was received for this work. However, in lines 130, 495, and 521, the author (s) acknowledge the financial support from PEPFAR through the CDC.

Abstract:

In the methods section, the phrase “returned to both sites and clients” (line 43) is ambiguous and could be misinterpreted as a standard practice or a primary outcome of the study. Based on the manuscript context, it seems results were made available to clients at follow-up visits as part of the study protocol. Consider revising the sentence to reflect the availability of results to clients at scheduled study follow-up visits as part of a supervised counselling process.

The phrase “health staff” in the sentence “four focus group discussions (FGDs) with health staff explored perceptions of POC-RTRI implementation” (lines 45-46) is broad and could include anyone from administrators to laboratory technicians. Please consider specifying the cadres who participated in the FDGs. For consistency and transparency, please ensure to explicitly state the cadres in the Methods section under “Qualitative Data Collection and Analysis”.

Discrepancy in the details in the abstract and results sections: In the abstract (lines 49-50, it states: “The POC-RTRI identified fewer recent infections than laboratory RTRO (85% vs 100.0%). However, in the main results (lines 264-275), the concordance table shows 27 recent at POC vs 35 recent at the Central laboratory, which is 77.1%, not 85%. Please clarify this discrepancy and ensure consistency between the abstract and the results sections. Additionally, the abstract reports 96.0 agreement between POC and laboratory RTRI, while Table 1 shows an agreement of 96.5. These likely reflect rounding/precision differences but require harmonization. Please consider providing an accurate agreement.

The Kappa value in the abstract (0.821) matches the main text Kappa for N=317 (0.812 on page 15 is likely a typo; should it be 0.821 as in the abstract?) Please clarify.

Methods:

Sample size: The methods section in the abstract and main text do not state the study’s sample size, which is a standard and necessary element for readers to assess the scale of the work. The sample size is first explicitly mentioned in the results section, on line 243 of the manuscript. Please consider adding the total number of enrolled participants for completeness.

Laboratory and test procedures: While the manuscript notes that telemonitoring was used to clarify faint band lines (line 190), the specific decision rule or visual threshold for interpreting faint Long Term lines is not described. To ensure methodological reproducibility and clarify the source of interpretation errors, please explicitly state the criteria used to determine whether a faint LT was recorded as present (long-term) or absent (recent). For example, was any visible line considered positive, or was a minimum threshold applied?

Data availability statement: Please consider depositing your data in a repository to obtain a citable DOI for the dataset linked to your manuscript. You can indicate the DOI in the statement, “De-identified and aggregated study data will be made publicly available through Data Dryad (doi: [pending]).

Results: Please consider expanding Table 3 to include the positive and negative predictive values that would help answer the question, “If this test result shows that the person was infected recently, how likely is that to be true?

Discussion: Consider linking the high false recent rate to the sensitivity analysis, which shows near-perfect specificity when excluding VL.
